# Gut microbiome and metabolome in aneurysm rat with hypertension after ginsenoside Rb1 treatment

**DOI:** 10.3389/fphar.2023.1287711

**Published:** 2023-11-23

**Authors:** Zhaobin Zeng, Haibin Wang, Renhui Yi, Jianyun Lou, Shuting Wen, Zheng Hu

**Affiliations:** First Affiliated Hospital of Gannan Medical University, Ganzhou, China

**Keywords:** microbiome, hypertension, ginsenoside, aneurysm, metabolome

## Abstract

**Introduction:** Hypertension is a well-known risk factor for aneurysms, as high blood pressure can worsen the development and rupture of aneurysms. Ginsenoside, derived from ginseng and widely used in traditional herbal medicine, is believed to have antihypertensive properties. Recent research has also shown a connection between gut microbiota and various diseases, including hypertension. However, the relationship between ginsenosides, gut microbiota, blood pressure, and intracranial aneurysms needs further exploration.

**Methods:** In this study, a rat model was used to investigate the effects of ginsenosides on both blood pressure and intracranial arteries. Comparative analysis was conducted, and 16S rRNA sequencing was employed to identify marker genera within the gut microbiota. Metabolites were also analyzed to uncover potential mediators of blood pressure regulation.

**Results and Discussion:** The results of this study revealed that ginsenosides, particularly ginsenoside Rb1, demonstrated positive effects in reducing both blood pressure and the development of intracranial aneurysms in rats. Furthermore, the analysis of gut microbiota showed that certain genera, including *Clostridium*, *Roseburia*, *Ruminococcus*, and *Treponema*, were significantly influenced by ginsenoside treatment. Several metabolites, such as behenic acid, N-Acetylserotonin, Prostaglandin F2a, and Vitamin D2, were also detected, all of which play a role in regulating blood pressure. These findings provide valuable insights into the potential benefits of ginsenosides in hypertension and atheroma development. Furthermore, they suggest a possible link between ginsenosides, gut microbiota, and blood pressure regulation. Further research is needed to fully understand the mechanisms underlying these effects and to determine the clinical implications for treating hypertension and reducing the risk of aneurysm development.

## Introduction

Intracranial aneurysm (IA) is a localized, aneurysmal dilatation of an intracranial artery, and it is a serious cerebrovascular disease with high morbidity and mortality (Sheinberg et al., 2019; Koester et al., 2023; Reddy and Masoud, 2023). Effective methods to stabilize and prevent IA rupture are currently lacking, and conservative treatment is most commonly employed for IA patients. The main pathological features of IA include vascular endothelial dysfunction, intimal hyperplasia, destruction of the extracellular matrix, and loss of arterial wall integrity due to inflammatory responses.

Hypertension is one of the significant risk factors for IA (Del Brutto et al., 2017; Lin et al., 2023). IA refers to the condition in which the arterial wall appears locally dilated or bulging, potentially leading to rupture and bleeding. Hypertension increases the risk of IA by exerting excessive pressure on the arterial wall. This pressure-induced structural damage to the arterial wall increases vessel wall fragility, thereby promoting the formation and rupture of IA (Shang et al., 2023). Moreover, long-term hypertension may contribute to arterial wall thickening, reduced elasticity, and atherosclerosis, further exacerbating the development of IA.

Ginsenosides are active ingredients extracted from ginseng and are widely used in traditional herbal medicine (Wang et al., 2022; Fan et al., 2023). Studies have shown that ginsenosides possess various pharmacological effects, including blood pressure regulatory. In the context of hypertension treatment, ginsenosides are believed to exhibit antihypertensive effects (Lee et al., 2020; Karmazyn and Gan, 2021). They act through various mechanisms, such as promoting the synthesis and release of nitric oxide (NO), improving vascular endothelial function, dilating blood vessels, and enhancing vascular elasticity, ultimately resulting in reduced blood pressure. Additionally, ginsenosides can inhibit sympathetic nerve activity, alleviate cardiac load, and exert protective effects on the heart and blood vessels. Ginsenosides have been found to inhibit the NF-κB signaling (Qin et al., 2019; Qi et al., 2022; Wang et al., 2023). Several studies have shown that ginsenosides can inhibit the activation of the NF-κB signaling pathway through different mechanisms (Jang et al., 2023). First, ginsenosides can inhibit the degradation of I kappa B proteins, thereby blocking the process of NF-κB entry into the nucleus. In addition, ginsenosides inhibit the DNA-binding ability of NF-κB, reducing its transcriptional regulation of specific genes. These effects enable ginsenosides to inhibit the expression of genes downstream of NF-κB, including inflammatory factors, apoptosis-related factors and immune response factors. However, it remains unclear if ginsenosides can reduce the risk of IA by modulating blood pressure, as studies in this area are still inconclusive.

Gut microbiome, symbiotically coexist with the human body and significantly influence human health (Jia et al., 2022; Jia et al., 2023; Xia, 2023). Research has linked the gut microbiome to various diseases, such as obesity, and hypertension. The impact of ginsenosides on gut microbiome has garnered significant attention. Several studies have suggested that ginseng and ginsenosides may influence the composition and function of gut microbiome (Santangelo et al., 2019; Yang et al., 2020; Chen et al., 2022). The active constituents within ginseng and ginsenosides exhibit antimicrobial effects in the intestinal tract, inhibiting the growth of harmful bacteria. Furthermore, they likely modulate intestinal microorganism metabolism, thereby influencing the gut microbiome. For instance, ginsenosides can alter the metabolite profiles of gut microbiome, affecting energy metabolism and nutrient absorption. Additionally, certain studies have indicated that ginseng and ginsenosides can promote the proliferation of probiotic bacteria such as *bifidobacteria* and *lactobacilli*, which play a crucial protective role in maintaining intestinal balance, as well as promoting nutrient absorption and digestion. Nevertheless, it should be noted that numerous unknown factors still surround the effects of ginseng and ginsenosides on gut microbiome. As such, a definitive understanding of the association between ginsenosides, gut microbiome, blood pressure, and intracranial aneurysms is presently lacking.

Therefore, in this study, we explored the changes in blood pressure, gut microbiome and peripheral blood metabolome in aneurysmal rats under ginsenoside treatment by using a rat model to preliminarily investigate the relationship between them.

## Materials and methods

### Animals and models

Eighteen male SD rats (200–250 g) were randomly divided into four groups: the model group, the amlodipine group, the ginsenoside group, and the control group of WKY rats. Animals in the model and treatment groups were anesthetized and underwent an abdominal incision, and the posterior branches of the renal arteries were isolated bilaterally using a microscope and then electrocoagulated by electrocoagulation. One week after surgery, the left common carotid artery was then electrocoagulated and severed. The rats in the treatment group were given 60 mg/kg-d of ginsenoside Rb1 per day, which was dissolved in saline to formulate the appropriate concentration and given by intraperitoneal injection, and body weight measurements were taken once a week, and the dosage was adjusted according to body weight. The control group was given the same normal feed after the simulated surgery. Twelve weeks after surgery, the animals were executed by perfusion with 4% paraformaldehyde solution, followed by craniotomy to remove the brain, and the ring of arteries at the base of the skull and its main branches were carefully observed under a dissecting microscope and stripped off, and finally stored at 4°C for spare parts.

### Blood pressure measurement

Arterial blood pressure was measured in different groups of rats using the tail-cuff method before treatment and at the 12th week of treatment. Measurements were made in a quiet place with few people, keeping the room temperature between 25°C and 26°C. The systolic blood pressure of the tail artery of rats in the awake state was measured using a BP-98A rat non-invasive sphygmomanometer, and each measurement was averaged three times.

### Sample collection and DNA extraction

Fresh feces were placed in a special fecal preservation tube in the early morning in the fasting state. The tubes were then quickly placed in a tank of liquid nitrogen for snap freezing, and stored in a −80°C refrigerator. DNA was extracted by commercial kit (TIANamp Stool DNA kit, Beijing Tiangen Biochemical Technology Co., Ltd.), and total DNA quality control was performed by NanoDrop (ThermoFisher, United States) and 1% agarose gel electrophoresis.

### Primer design, PCR amplification, and sequencing

The V3-V4 region of 16S rDNA was selected, and the universal primers were designed as 341F and 806R. The primer design was as follows: forward primer (5′-3′): CCTACGGGRSGCAGCAG (341F); reverse primer (5′-3′): GGACTACVVGGGGTATCTAATC (806R). PCR amplification was carried out with PCR kit (Roche, Switzerland) using diluted genomic DNA as template After passing the quality control, the libraries were quantified using Qubit. Sequencing was carried out with Illumina Miseq PE250 by Biotree company. Raw data were deposited in NCBI database as PRJNA1010153.

### OTU analysis, diversity analysis

To facilitate the subsequent analysis of species diversity, clustering of long Reads was performed with 97% similarity using Usearch software, and the sequences were divided into operational taxonomic units (OTUs) based on similarity. The diversity indices of the samples were calculated using QIIME software and the corresponding dilution curves were generated. Values of *p* diversity indices were calculated using an iterative algorithm using QIIME software and differences were calculated under weighted species taxonomic abundance information and unweighted species taxonomic abundance information, respectively.

### Significant difference analyses

Linear discriminant analysis of effect sizes (LEfSe) was used to estimate the magnitude of the effect of different component abundances on the effect of differences and to identify communities or species that significantly differed in the delineation of the samples. LEfSe Tools was used to perform this analysis. Significant difference analyses were performed using the rank sum test to identify species that produced significantly different effects on group delineation.

### Metabolome

Metabolite extraction, Chromatographic conditions, Mass spectrometry conditions were performed by Biotree company.

## Results

### Pathological changes in IA rats after ginsenoside Rb1 treatment

**FIGURE 1:**
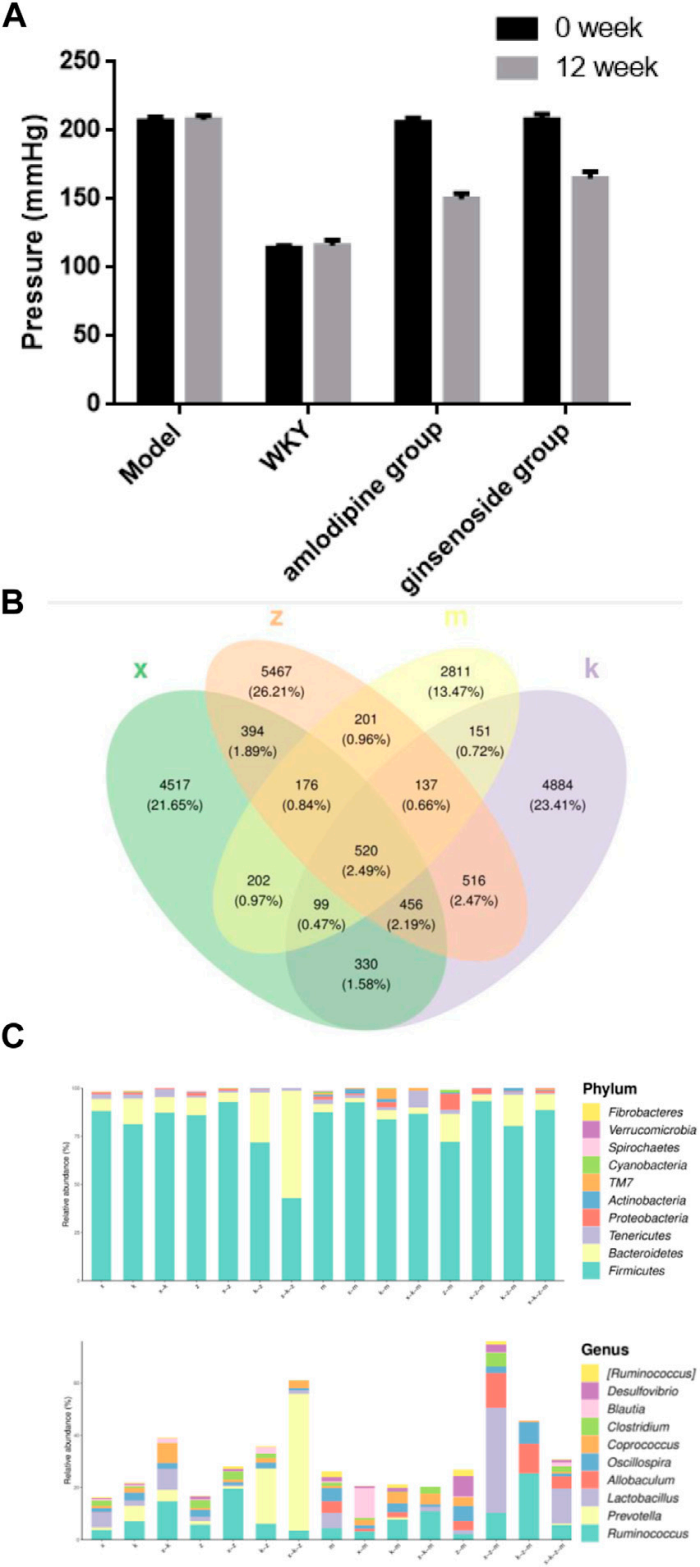
The composition of gut microbiome in aneurysm rat with hypertension after ginsenoside Rb1 treatment. **(A)** Ginsenoside Rb1 treatment reduce the hypertension of aneurysm rat. **(B)** Venn diagram of gut microbiome OTU. x, WKY group; m, amlodipine group; k, model group; z, ginsenoside group. **(C)** Represent Phylum and Genus of gut microbiome in different group. Note that four major phyla: *Firmicutes*, *Bacteroidetes*, *Tenericutes*, and *Proteobacteria*; four dominant genera were identified: *Ruminococcus*, *Prevotella*, *Lactobacillus*, and *Allobaculum*.

The effects of ginsenoside Rb1 treatment on IA rats were investigated, specifically focusing on blood pressure regulation and arterial abnormalities. After 12 weeks of treatment, both the ginsenoside Rb1 and amlodipine groups exhibited a more significant reduction in blood pressure compared to the model group. The decrease in blood pressure was not as pronounced in the ginsenoside Rb1 group as in the amlodipine group, which served as the positive control drug. While both high-dose ginsenoside Rb1 and amlodipine effectively reduced blood pressure in the spontaneously hypertensive rats (SHR), the ginsenoside Rb1 group did not fully restore blood pressure to the level of normotensive WKY rats (Figure 1A).

### General results of IA rat gut microbiome after ginsenoside Rb1 treatment

In this study, an average of over 5,000 operational taxonomic units (OTUs) were identified per sample. The OTU Venn diagram illustrates the overlap and uniqueness of OTUs among the groups. Among the total OTUs, 520 OTUs were found to be shared by all four groups, while 4,884 were unique to the modeling group, 2,811 were exclusive to the amlodipine group, 4,517 were specific to the WKY group, and 5,467 were exclusive to the ginsenoside group (Figure 1B).

To gain further insights into the taxonomic distribution of species within each region of the Venn diagram, the number of corresponding amplicon sequence variants (ASVs) or OTUs in each region was analyzed at the phylum and genus level, and the results were presented using bar charts (Figure 1C). The analysis revealed that the gut microbiome predominantly consisted of four major phyla: *Firmicutes*, *Bacteroidetes*, *Tenericutes,* and *Proteobacteria*. At the genus level, four dominant genera were identified: *Ruminococcus*, *Prevotella*, *Lactobacillus*, and *Allobaculum*.

**FIGURE 2:**
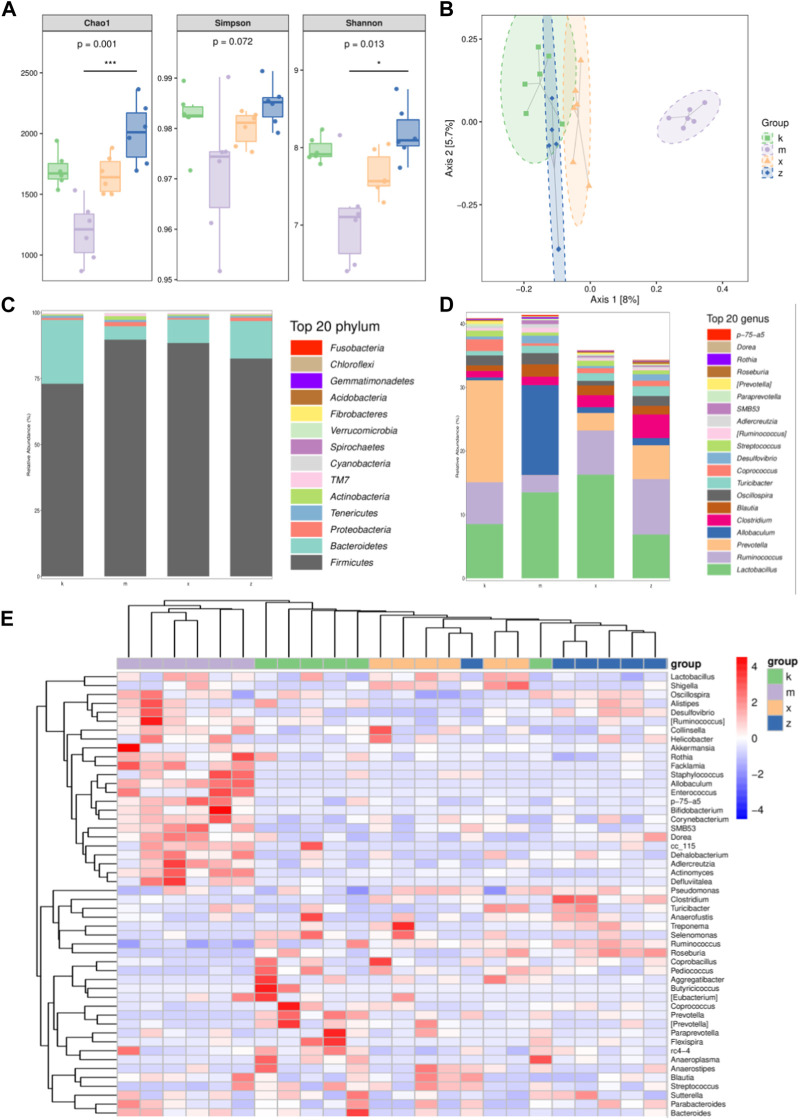
The different characteristics of gut microbiome in aneurysm rat with hypertension after ginsenoside Rb1 treatment. **(A)** The Chao1, Simpson, Shannon index. **(B)** PcoA analysis. **(C,D)** Top 20 phylum and genus with different abundance in gut microbiome. Note that four phyla: Firmicutes, Bacteroidetes, Tenericutes, and Proteobacteria. Additionally, four genera, namely, Ruminiococcus, Prevotella, *Lactobacillus*, and Allobaculum. **(E)** Heatmap of differential genus.

To further investigate changes in the diversity of the gut microbiome within each group, several indexes were calculated. These indexes describe both the species richness and diversity of the gut microbiome (Figure 2A). Specifically, the Shannon index and Simpson index reflect community diversity, while the Chao1 index reflects community richness. The findings demonstrated that the Chao1 index decreased in the amlodipine group. Conversely, the Chao1 index increased in the ginsenoside Rb1 group, indicating an increase in the abundance of its flora. Similarly, the Shannon index and Simpson index followed the same trend as the Chao1 index in all four groups. This indicates that the amlodipine group exhibited a decrease in flora diversity, while the ginsenoside Rb1 group showed an increase in flora diversity.

In the study, the principal coordinate analysis (PCoA), a dimensionality reduction technique, was used to visualize the differences between samples in a two-dimensional coordinate system based on their similarity distance (Figure 2B). The distance between points represented the magnitude of the differences between the samples. The results of the analysis revealed distinct clustering of the four sample groups. Particularly, the amlodipine group exhibited the most isolated distribution, indicating a difference in the composition of the gut microbiome compared to the other groups.

In analyzing the taxonomic distribution of species across different groups of samples, the results revealed that the intestinal flora primarily consisted of four phyla: *Firmicutes*, *Bacteroidetes*, *Tenericutes*, and *Proteobacteria*. Additionally, four genera, namely, *Ruminiococcus*, *Prevotella*, *Lactobacillus*, and *Allobaculum*, were predominant in the samples (Figures 2C, D). To delve further into the differences in species composition between samples and present trends in species abundance distribution, heat map analysis was employed (Figure 2E). The four groups of samples exhibited distinct clustering based on the distribution of species abundance. Specifically, all six samples in the amlodipine group formed a single cohesive cluster, while one sample each from the model and WKY groups, as well as one sample from the model and ginsenoside Rb1 groups, deviated from their respective clusters. Moreover, the ginsenoside Rb1 group exhibited a greater similarity to the WKY group. In terms of specific genera, the abundance of *Lactobacillus, Shigella*, *Allobaculum*, and *Actinomyces* was higher in the amlodipine group, forming a distinct cluster. On the other hand, *Pseudomonas*, *Clostridium*, *Turicibacter*, *Anaerofustis*, *Treponema*, *Selenomonas*, *Ruminococcus*, and *Roseburia* exhibited higher abundance in the ginsenoside Rb1 group and clustered together. Meanwhile, *Coprobacillus*, *Pediococcus*, *Aggregatibacter*, *Butyricicoccus*, *Eubacterium*, *Coprococcus*, *Prevotella*, *Paraprevotella*, *Flexispirarc4-4*, *Anaeroplasma*, *Anaerostipes*, and other genera displayed higher abundance in the model group and clustered together.

### Differential genus in gut microbiome

**FIGURE 3:**
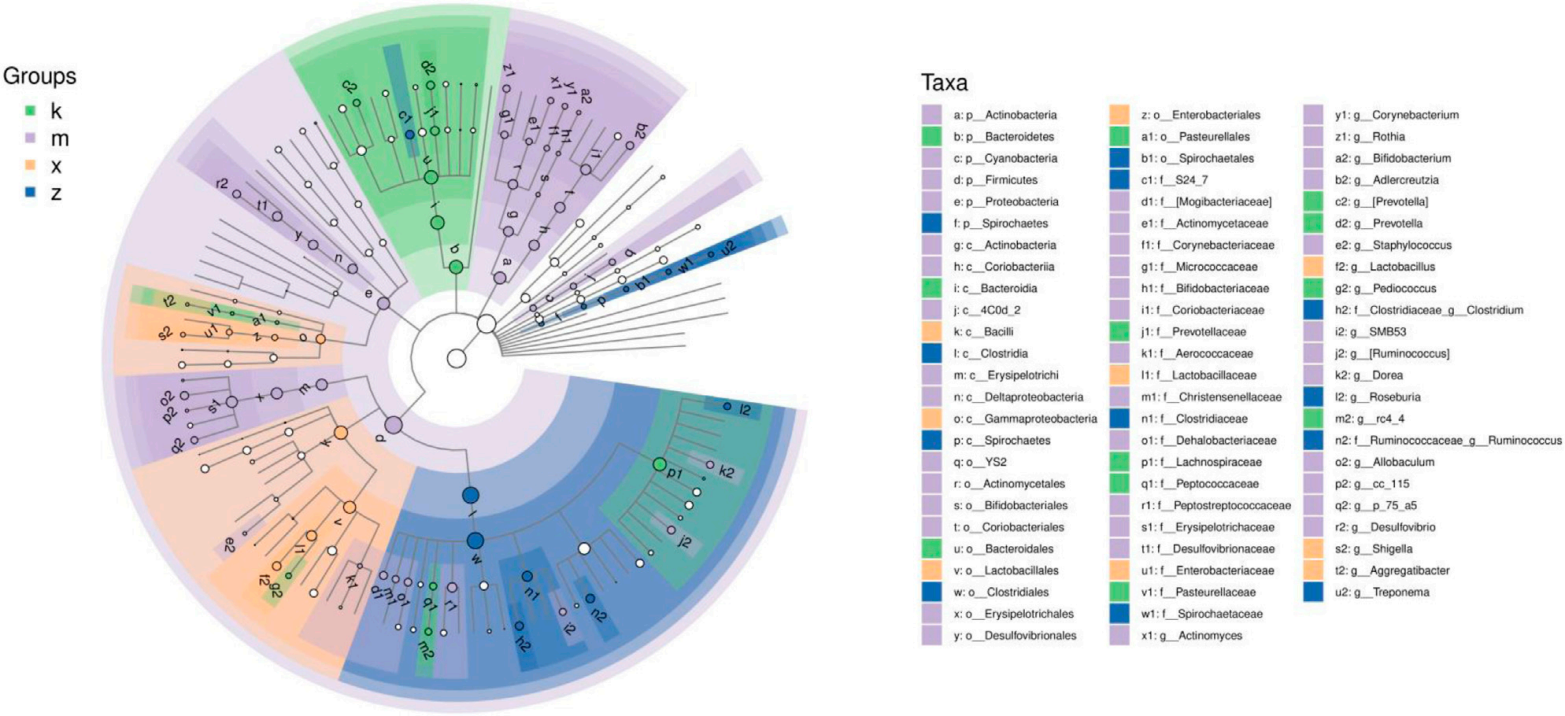
LDA Effect Size analysis of gut microbiome in aneurysm rat with hypertension after ginsenoside Rb1 treatment.

We performed LEfSe (LDA Effect Size) analysis to identify robust differential species between subgroups (Figure 3). The analysis revealed the following flagged genera in the modeled array: *Prevotella*, *Pediococcus*, and *rc4_4*. In the amlodipine group, the flagged genera were *Actinomyces*, *Corynebacterium*, *Rothia*, *Bifidobacterium*, *Adlercreutzia*, *Staphylococcus*, *SMB53*, *Ruminococcus*, *Dorea*, and *Allobaculum*. In the WKY group, *Lactobacillus*, *Shigella*, and *Aggregatibacter* were identified as the marker genera. In the ginsenoside Rb1 group, the marker genera were *Clostridium*, *Roseburia*, *Ruminococcus*, and *Treponema*.

**FIGURE 4:**
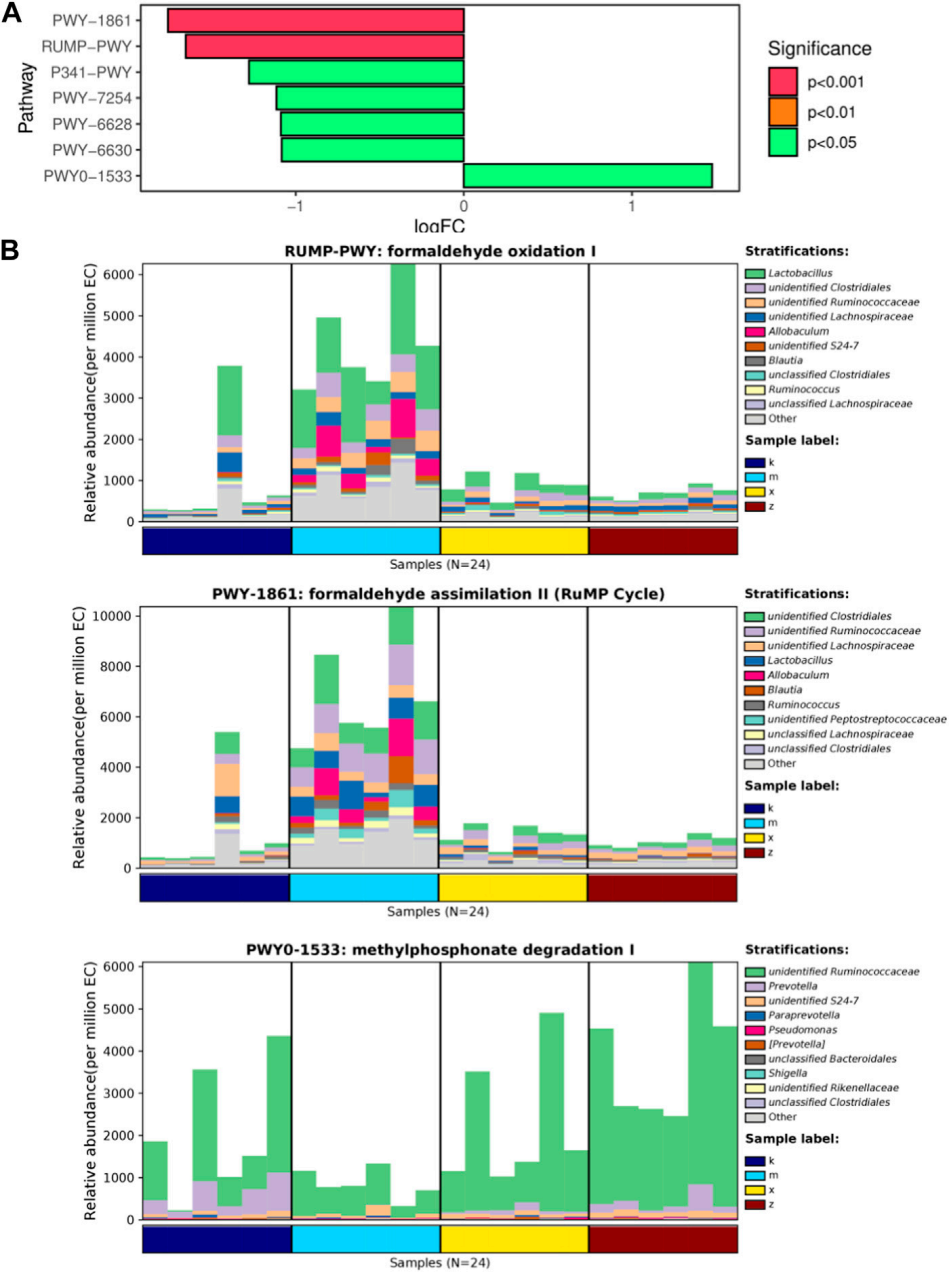
KEGG pathway analysis of differential genus in gut microbiome. **(A)** Top enriched pathways. **(B)** Represent pathways as formaldehyde oxidation I, RuMP Cycle, methylphosphonate degradation I.

After analyzing the abundance data of bacterial groups in each metabolic pathway, we can focus on identifying the metabolic pathways that exhibit significant differences among the groups (Figure 4A). In comparison to the ginsenoside Rb1 group, only the WKY group displayed distinct enrichment in metabolic pathways like formaldehyde oxidation I, formaldehyde assimilation II (RuMP cycle), and methylphosphonate degradation I. Furthermore, the WKY group demonstrated significant dissimilarity when compared to the ginsenoside Rb1 group (Figure 4B). Notably, these pathways involve the genera *Lactobacillus*, *Clostridiales*, and *Ruminococcaceae*, respectively.

### Difference in metabolome

**FIGURE 5:**
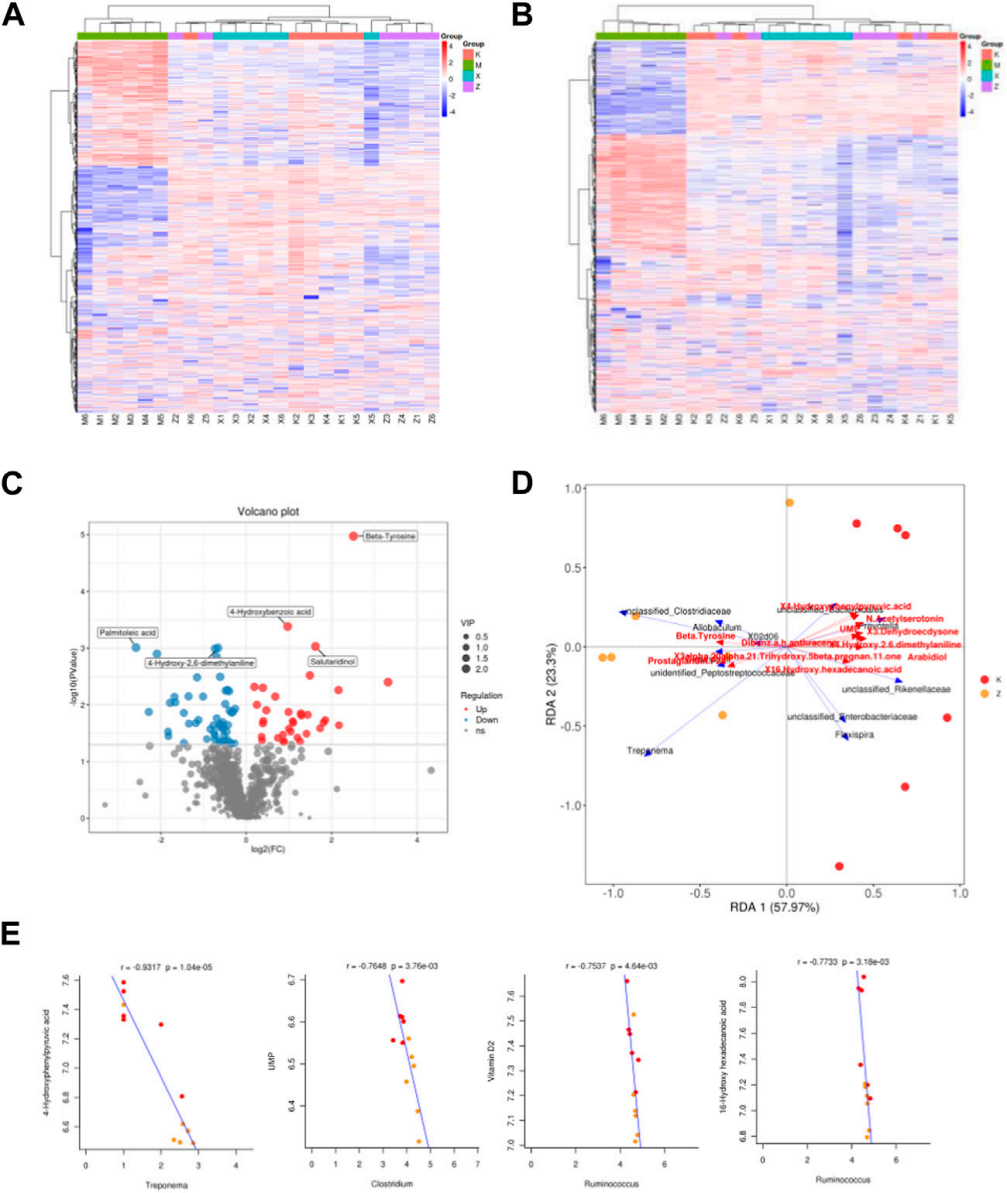
Different metabolome in aneurysm rat with hypertension after ginsenoside Rb1 treatment. **(A,B)** Heatmap of differential metabolites in positive and negative ions. x, WKY group; m, amlodipine group; k, model group; z, ginsenoside group. **(C)** Volcano plot reveals differential metabolites between model and ginsenoside group. **(D)** PcoA analysis of differential metabolites and gut microbiome. **(E)** Correlation between metabolites and gut microbial species.

In order to analyze the flora at the genus level, variables with significant correlation coefficients (*p* < 0.05) were carefully selected for the construction of the RDA analysis plot (Figures 5A, B). In this plot, an acute angle between two variables signifies a positive correlation, indicating synergistic effects, while an obtuse angle reflects a negative correlation, suggesting antagonistic effects. Comparing the model group to the ginsenoside Rb1 group, several genera were found to be associated with specific metabolites. These genera included *Treponema*, unclassified_Rikenellaceae, unidentified_Peptostreptococcaceae, Allobaculum, unclassified_Clostridiaceae, *Clostridium*, 02d06, unclassified_Enterobacteriaceae, Flexispira, Prevotella, unclassified_Bacteroidales. The metabolites associated with these genera comprised of 4-Hydroxyphenylpyruvic acid, Beta-Tyrosine, Brassinolide, 3-Dehydroecdysone, Behenic acid, Arabidiol, UMP, and other unidentified compounds (Figures 5C–E).

Representative genera of the ginsenoside Rb1 group *Clostridium*, *Ruminococcus*, and *Treponema* have significantly related metabolites, including 4-Hydroxyphenylpyruvic acid, UMP, and Vitamin D2 (Figure 4).

## Discussion

Hypertension, characterized by excessive blood pressure in the arteries, has a complex mechanistic impact on the development and progression of aneurysms (Addis et al., 2023; Rega et al., 2023; Sharma et al., 2023). Several potential correlates have been identified. First, sustained high blood pressure weakens the arterial wall by increasing pressure load, rendering it more susceptible to aneurysm formation. Second, hypertension can lead to vascular inflammation and damage, disrupting arterial wall structure and promoting aneurysm development. Third, hypertension causes abnormal blood flow, such as increased velocity or turbulence, generating shear forces that damage the arterial wall and facilitate aneurysm formation. Fourth, hypertension may induce abnormal proliferation of vascular smooth muscle cells, which can result in arterial wall thinning and increased structural instability, promoting aneurysm formation.

While hypertension plays a crucial role in aneurysm formation, other factors, such as genetic factors, structural abnormalities of the vessel wall, and tumor vascular supply, may also contribute to aneurysm development and progression. Studies examined the effects of ginsenoside Rb1 treatment on hypertension and aneurysm and found that it relieved both conditions, consistent with previous published studies (Zhang et al., 2015; Wang et al., 2020). Ginsenoside, an active ingredient derived from ginseng, is widely used in hypertension treatment. Its mechanism of action may involve vasodilation, anti-inflammatory effects, reducing burden on the heart, and antioxidant properties.

Recent studies have revealed that ginsenosides can influence the intestinal flora in hypertensive patients (Yang et al., 2021; Zhou et al., 2023). The gut flora refers to microbial communities in the human intestinal tract, which significantly impact human health. Hypertensive patients exhibit dysbiosis, indicating differences in intestinal flora compared to normal individuals. Ginsenosides, known for their anti-inflammatory and antioxidant effects, can modulate the immune system, metabolic status, and composition and function of the intestinal flora, thereby improving hypertension. They can increase the abundance of beneficial flora, such as lactobacilli and *bifidobacteria*, while decreasing harmful bacteria (Han et al., 2020). This alteration affects signaling pathways related to gut health, immune responses, and inflammation levels, potentially benefiting hypertension. Nevertheless, further research is necessary to validate the effects of ginsenosides on gut flora and their association with hypertension treatment. Additionally, the complex and diverse nature of the intestinal flora suggests the involvement of other factors in the mechanism of action of ginsenosides in hypertension treatment. Despite the lack of definitive findings, aneurysms are associated with certain cardiovascular diseases. Therefore, it can be speculated that an imbalance in the intestinal flora may have some connection with aneurysms. Further investigations are required to explore this hypothesis.

In a study, the ginsenoside Rb1 group exhibited marked increases in the genera *Clostridium*, *Roseburia*, *Ruminococcus*, and *Treponema* (Wang et al., 2021). Another group showed that losartan, in addition to reducing blood pressure, increased the levels of *Aristophanes*, *Lactobacillus*, and *Butyric acidophilus*, while decreasing *Ruminococcaceae*, *Streptococcus*, and *Turbotobacteria* (Luo et al., 2023). Furthermore, a survey of the fecal microbiota demonstrated a positive association between specific *Vibrio* lachnosus spp. and high-density lipoprotein cholesterol but a negative association with systolic and diastolic blood pressure (McPherson et al., 1991). Although an 8-week intervention did not significantly alter gut microbiota composition, an induction of Prevotella was observed in the intervention group. Additionally, *Treponema pallidum* was positively correlated with blood pressure. Another study exploring the effects of a low-carbohydrate diet and exercise training found positive correlations between changes in *Ruminococcus spp*, *Escherichia coli* spp., *Rosella spp*, and blood pressure (Sun et al., 2022). Moreover, an engineered probiotic, *Clostridium butyricum* pMTL007 GLP 1, improved blood pressure by producing GLP 1 and influencing the gut microbiota in a rat model of hypertension (Wang et al., 2023). These findings indicate a complex regulatory mechanism connecting the marker genera observed in the ginsenoside Rb1 group and blood pressure.

On the other hand, we have identified a series of metabolites that are associated with the abundance of gut bacteria in the ginsenoside Rb1 group. Several metabolites have been extensively reported as strongly correlated with blood pressure. Notably, one study has confirmed the association between lauric acid found in circulating saturated fatty acids and the risk of pregnancy-induced hypertension (Li et al., 2020). Additionally, N-acetyltryptophan has been found to possess antioxidant effects (Oxenkrug, 2005). Furthermore, in a rat model of bacterial lipopolysaccharide-induced hypotension, it has been observed that N-acetyltryptophan may counteract hypotension by inhibiting tetrahydrobiopterin biosynthesis (Klemm et al., 1993). Vitamin D2, whose body levels have been documented to correlate with blood pressure in humans (Pedersen et al., 2022). Moreover, prostaglandin F2a has been suggested to potentially regulate blood pressure through calcium and PKC signaling (Plewes et al., 2023). Importantly, prostaglandin F2a itself holds promise as a potential therapeutic drug.

In conclusion, hypertension has a multifaceted impact on aneurysm development, involving arterial wall weakening, vascular inflammation, altered blood flow, and abnormal vascular smooth muscle cell proliferation. While ginsenosides have shown potential in alleviating hypertension and aneurysms, further research is needed to elucidate their precise therapeutic effects. Additionally, ginsenosides have been found to modulate the intestinal flora in hypertensive patients, potentially influencing hypertension through immune modulation and inflammation regulation. Although the specific role of gut flora in aneurysm treatment remains unclear, an association between intestinal dysbiosis and aneurysms can be hypothesized. However, comprehensive studies are required to validate this hypothesis.

## Data Availability

The datasets presented in this study can be found in online repositories. The names of the repository/repositories and accession number(s) can be found below: NCBI BioProject (https://www.ncbi.nlm.nih.gov/bioproject/), PRJNA1010153.
